# Sex differences in acupuncture effectiveness in animal models of Parkinson's disease: a systematic review

**DOI:** 10.1186/s12906-016-1405-5

**Published:** 2016-11-03

**Authors:** Sook-Hyun Lee, Maurits van den Noort, Peggy Bosch, Sabina Lim

**Affiliations:** 1 Department of Applied Korean Medicine, Graduate School, Kyung Hee University, Seoul, Republic of Korea; 2 Research Group of Pain and Neuroscience, WHO Collaborating Center for Traditional Medicine, East–west Medical Research Institute, Kyung Hee University, Seoul, Republic of Korea; 3 Donders Institute for Brain, Cognition and Behaviour, Radboud University, 6525 HR Nijmegen, The Netherlands; 4 Department of Meridian & Acupoint, College of Korean Medicine, Kyung Hee University, 26 Kyungheedae-ro, Dongdaemun-gu, Seoul, 130-70102447 Republic of Korea

**Keywords:** Electro-acupuncture, Manual acupuncture, Bee-venom acupuncture, C57/BL6, Acupuncture point

## Abstract

**Background:**

Many animal experimental studies have been performed to investigate the efficacy of acupuncture in Parkinson’s disease (PD). Sex differences are a major issue in all diseases including PD. However, to our knowledge, there have been no reviews investigating sex differences on the effectiveness of acupuncture treatment for animal PD models. The current study aimed to summarize and analyze past studies in order to evaluate these possible differences.

**Method:**

Each of 7 databases (MEDLINE, EMBASE, the Cochrane Library, 3 Korean medical databases, and the China National Knowledge Infrastructure) was searched from its inception through March 2015 without language restrictions.

**Results:**

We included studies of the use of acupuncture treatment in animal models of PD. A total of 810 potentially relevant articles were identified, 57 of which met our inclusion criteria. C57/BL6 mice were used most frequently (42 %) in animal PD models. Most of the studies were carried out using only male animals (67 %); only 1 study (2 %) was performed using solely females. The further 31 % of the studies used a male/female mix or did not specify the sex.

**Conclusions:**

The results of our review suggest that acupuncture is an effective treatment for animal PD models, but there is insufficient evidence to determine whether sex differences exist. Future studies of acupuncture treatment for PD should use female animal models because they reflect the physiological characteristics of both males and females to fully evaluate the effect and the safety of the treatment for each sex.

**Electronic supplementary material:**

The online version of this article (doi:10.1186/s12906-016-1405-5) contains supplementary material, which is available to authorized users.

## Background

Parkinson’s disease (PD) is a progressive neurodegenerative disease caused by the loss of dopaminergic neurons in the substantia nigra [1]. PD usually occurs in individuals over 50 years of age, and its incidence and prevalence increases among individuals approximately 60 years of age and older. PD has become more common due to the rapid aging of human populations around the world [2]. Epidemiological studies have reported that the incidence of PD is 1.5–2 times higher in men than in women, and the onset of symptoms may occur later in women due to the neuroprotective effects of estrogen [3]. For the disease manifestations of PD, women have higher Unified Parkinson’s Disease Rating Scale (UPDRS) motor scores, but present with dyskinesia, tremor, and PD-related complications more often than men [4].

Because the FDA reported that eight out of ten new drugs that had been sold on the market were discontinued because they resulted in far more detrimental side effects in women, the sex perspective began to be discussed in many other fields as well [5]. Adverse drug reactions can be caused by the physiological difference between men and women, and women can be more vulnerable to a particular drug [6]. Because sex is often not considered an important variable in animal research with the exception of research related to features of a particular sex, such as reproduction and endocrine secretion, the overwhelming majority of experimental research uses only males and many studies do not even disclose the sex of the experimental animals. Basic research studies using cells in culture also often fail to present the sex of the organism from which the cell strain originated, but the results of such basic research has been applied generally to humans. Because medical research studies are performed primarily by male researchers [7–9], the research subjects are also mostly males [10–12], and there has been a tendency to be careless of females [13], which can aggravate treatment problems related to the physiological differences between men and women. The National Institutes of Health (NIH) requires applicants to report their cell and animal inclusion plans as part of the preclinical experimental design [14]. Therefore, studies are being performed to determine what sex differences need to be accounted for in preclinical and clinical stages, and the importance of the applying these principles is being highlighted [15].

PD treatment options include pharmacological treatment, non-pharmacological treatment, surgical therapy, and dopaminergic cell transplantation [15]. Acupuncture has long been employed for numerous disorders, and it has been traditionally used to relieve PD-related symptoms and to delay the clinical progression of PD symptoms [16]. We have reported that acupuncture exerts increased neuroprotective effects in regions including the substantia nigra, caudate, thalamus, and putamen in animal models of PD [17–20]. Acupuncture was also found to inhibit microglial activation, inflammation, and iron-related oxidative damage in PD [21].

Sex differences have emerged recently as an important issue, but sufficient efficacy tests for sex differences in acupuncture, as in preclinical studies for drug development, have not yet been performed. It is necessary to clarify efficacy differences according to sex in order to more effectively utilize acupuncture in clinical practice. Therefore, we carried out the present study to identify whether adequate research has been conducted so far to determine the sex differences in the efficacy of acupuncture. Specifically, we analyzed past studies of acupuncture treatment conducted in animal PD models, and determined whether the body of data was sufficient to determine the effects of sex differences on the effectiveness of acupuncture treatment. This review provides the basis for establishing whether future animal model studies are necessary to determine possible sex-related differences in the efficacy of acupuncture for PD.

## Methods

### Search methods for the identification of studies

The search was performed without restrictions on language or year of publication. We searched Medline, EMBASE, and the Cochrane Central Register of Controlled Trials from the inception of each database through March 2015. For Korean publications, we searched three Korean medical databases (Research Information Service System, National Discovery for Science Leaders, and OASIS). For Chinese articles, we searched the China National Knowledge Infrastructure. The keywords used for the search were the following: “Parkinson’s disease” OR “Parkinson” AND “acupuncture” OR “acupoints” OR “electroacupuncture” OR “electro-acupuncture” OR “auriculotherapy” OR “auriculoacupuncture” OR “bee venom acupuncture” in each database language. The search strategy was adjusted for each database.

### Inclusion/exclusion criteria

We included studies of the use of acupuncture treatment in animal PD models. Trials were excluded if the study designs did not evaluate the effectiveness of acupuncture in animal PD models, or if they reported insufficient data. No search restrictions on language or publication forms were imposed. During the first stage of selection/exclusion, titles and abstracts were analyzed, and literature that had no relevance to our study was excluded. The second stage of selection/exclusion involved analyzing the full text of particular studies, because it was impossible to determine the relevance of the studies based solely on the abstracts.

### Data extraction

Two reviewers (LSH and KJY) independently reviewed the data extracted from each article using a standardized data extraction form and reached consensus on all items. The extracted data included the type of animal PD models, the sex of the animal PD models, the methods used to induce PD, the types of acupuncture, the acupuncture points, and the effectiveness of the treatment.

## Results

### Study description

**Fig. 1:**
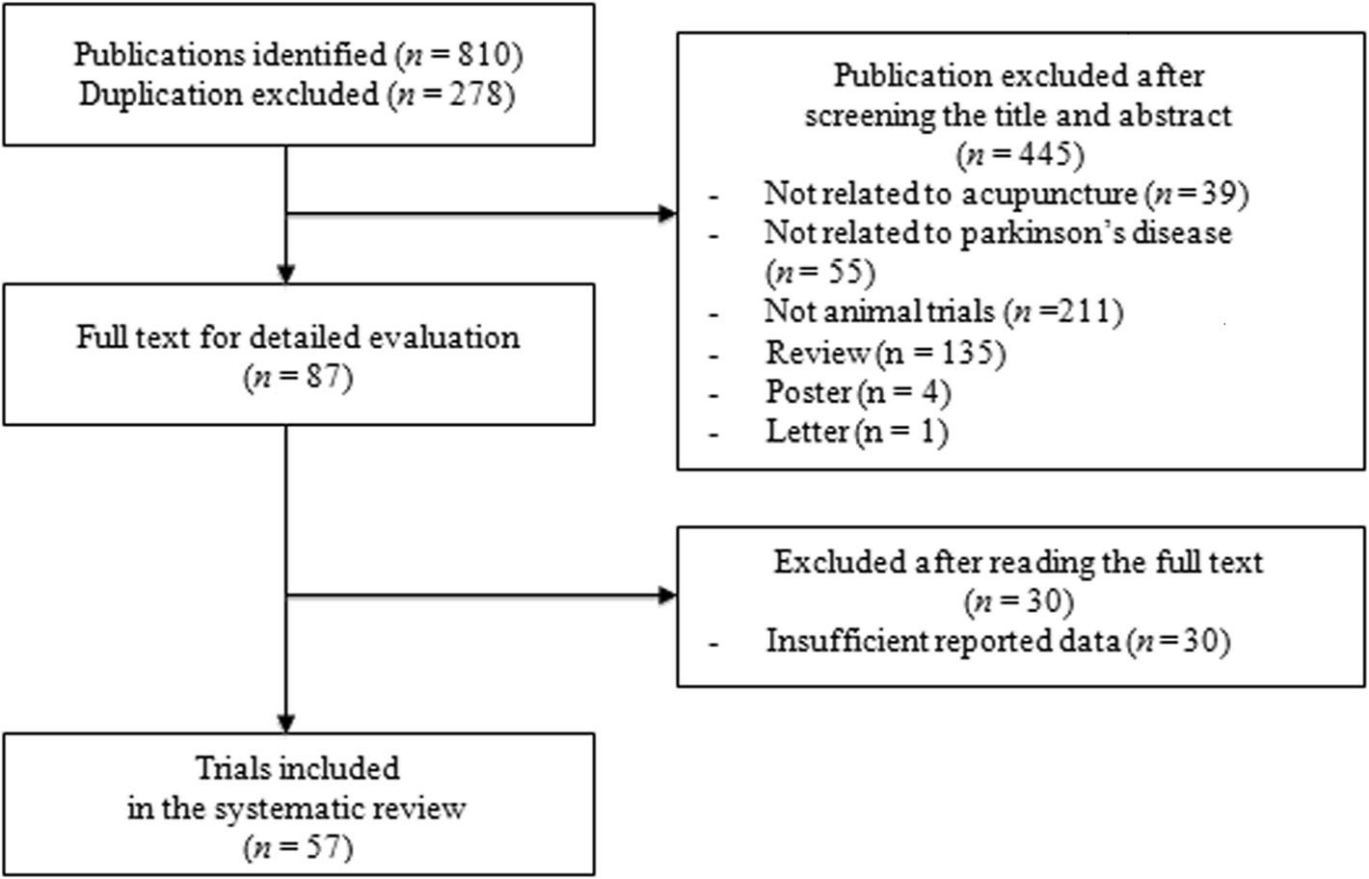
Flowchart of the study selection process

We identified 810 publications, 57 of which met the eligibility criteria (Fig. 1). The 57 articles were published from 1996 to 2014. The characteristics of the studies are summarized in Table 1 [7–12, 18, 19, 21–69].

**Table 1:** Summary of acupuncture for animal PD models

First author (year)	Type of animal PD models	Sex of animal PD models	Drugs used to induce PD	Types of acupuncture	Types of acupuncture points	Evaluation of the treatment effectiveness
Bai (2014a) [22]	Undefined	Undefined	6-OHDA	EA	GV20, EX-HN5	DA
Bai (2014b) [8]	Undefined	Male	6-OHDA	EA	GV20, EX-HN5	Caspase-3
Feng (2014) [10]	C57BL/6	Male	MPTP	MA	Undefined	Pole-climbing test, BDNF, TH, DA
Yeo (2013) [19]	C57BL/6	Male	MPTP	MA	GB34, LR3	TH, gene expression
Alvarez-Fischer (2013) [7]	C57BL/6	Male	MPTP	BV	Undefined	DA, DOPAC, IL-1β, IL-6, TNF-α, HVA, TH, rotational test
Ding (2013) [11]	SD	Male	6-OHDA	EA	LI4, LR3	nNOS, GFAP
Wang (2013a) [9]	SD	Male	Rotenone	EA	GV16, LR3	TH, COX-2
Wang (2013b) [23]	SD	Male	Rotenone	EA	GV16, LR3	TH, p-p38 MARK, COX-2
Wang (2013c) [12]	SD	Male	Rotenone	EA	GV16, LR3	TH, SOD, GSH, CAT, MDA
Wang (2013d) [24]	SD	Male	Rotenone	EA	GV16, LR3	UCH-L1, UBE1, Parkin, TH, α-synuclein
Ding (2012) [25]	SD	Male	6-OHDA	EA	LI4, LR3	TH, GFAP, PCNA
Huang (2012) [26]	ICR	Male	MPTP	EA	GB34	Lamp 1, α-synuclein
Lu (2012) [27]	C57BL/6	Male	MPTP	EA	GV20, GV16, GB34	Locomotor counts, swimming test, pole-climbing test
Guo (2012) [28]	SD	Male	6-OHDA	EA	GV20, GV16, GB34	GSH, SOD, MDA, GSH-Px
Yang (2011) [29]	C57BL/6	Male	MPTP	EA	PC7	Pole-climbing test, TH, DA, DOPAC, HVA
Choi (2011) [18]	C57/BL6	Male	MPTP	MA	GB34, LR3	TH, DAT, gene expression
Kim (2011) [30]	C57BL/6	Male	MPTP	BV	ST36	MAC-1, iNOS, TH
Du (2011) [31]	SD	Male	6-OHDA	EA	GV20, GV14	GABA, rotational test
Wang (2011) [32]	C57BL/6	Male	MPTP	EA	ST36, SP6	TH, DA, DOPAC, HVA, SOD, GSH, GSH-Px
Doo (2010) [33]	C57BL/6	Male	MPTP	BV	GB34	TH
Hong (2010) [34]	C57BL/6	Male	MPTP	MA	GB34	Gene expression
Jun (2010) [35]	C57BL/6	Male	MPTP	BV	BL23	TH, caspase-3, iNOS
Kim (2010) [36]	C57BL/6	Male	MPTP	EA	GB34, GB39	DA
Park (2010) [37]	C57BL/6	Male	MPTP	BV	GB39, LI11, BL23	TH, MAC-1, HSP70
Sun (2010) [38]	C57BL/6	Male	MPTP	MA	GV20, GV14	Pole-climbing test, TH, DA, DOPAC
Wang (2010a) [39]	Wistar	Undefined	6-OHDA	EA	GV16, LR3	TH, DA
Wang (2010b) [40]	Wistar	Undefined	6-OHDA	EA	GV16, LR3,CV4, ST36	GDNF
Wang (2010c) [41]	C57/BL6	Male	MPTP	MA	GV20, GV14	Pole-climbing test, TH, DA, NA, DOPAC, 5HIAA, 5HT
Yu (2010) [42]	Wistar	Male	6-OHDA	MA	GB34, LR3, ST36, SP10	Rotational test, SOD, GSH-Px, CAT, GSH, MDA
Huang (2010) [43]	Wistar	Male	6-OHDA	EA	LI4, LR3	Rotational test, BDNF, TrKB
Choi (2009) [21]	C57/BL6	Male	MPTP	MA	LR3, GB34	TH, DAT
Kim (2009) [44]	C57BL/6	Male	MPTP	BV	BL23	TH, MAC-1, HSP70
Wang (2009a) [45]	Wistar	Male, Female	6-OHDA	EA	GV20, EX-NH5	TH, BDNF
Wang (2009b) [46]	Wistar	Male, Female	6-OHDA	EA	GV20, EX-NH5	TH, DAT
Kim (2008) [47]	C57BL/6	Male	MPTP	MA	GB34	TH
Guan (2008) [48]	C57BL/6	Male	MPTP	EA	GV20	Fn
Wang (2008) [49]	Wistar	Male, Female	6-OHDA	EA	GV20, EX-NH5	TH
Jeon (2008) [50]	C57BL/6	Male	MPTP	EA	GB34, SI3, BL62, ST36	Pole-climbing test, TH, DA, BDNF
Xie (2007) [51]	Wistar	Undefined	6-OHDA	MA	GV20	Rotational test, MDA, NO, SOD
Kang (2007) [52]	C57BL/6	Male	MPTP	MA	GB34, LR3	TH, COX-2, iNOS, DA, DOPAC, HVA
Huang (2007) [53]	SD	Male	6-OHDA	MA	GB34, LR3	TH
Luo (2007) [54]	Wistar	Male, Female	6-OHDA	EA	GV20, EX-NH5	NOS
Wang (2007) [55]	SD	Male, Female	6-OHDA	MA	GV20, GV16, GB34	Rotational test, DA
Jin (2006a) [56]	Wistar	Male, Female	6-OHDA	EA	Undefined	GSH, GSH-Px,SOD, MDA, NOS
Jin (2006b) [57]	Wistar	Male, Female	6-OHDA	EA	Undefined	DA, HVA, DOPAC
Ma (2006) [58]	Wistar	Male, Female	6-OHDA	EA	GV16, LR3	Rotational test, DA
Tang (2006) [59]	C57BL/6	Male	MPTP	EA	LI4, LR3	BDNF
Wang (2006) [60]	SD	Male, Female	6-OHDA	EA	GV16, LR6	Glutamic acid
Kim (2006) [61]	C57BL/6	Male	MPTP	MA	LR8, LR4, LR2	TH
Kim (2005) [62]	SD	Undefined	6-OHDA	MA	ST36	Rotational test, TH
Ma (2005) [63]	Wistar	Male, Female	6-OHDA	EA	GV16, LR3	Rotational test, SOD, GSH, GSH-Px
Wang (2005) [64]	Wistar	Undefined	6-OHDA	MA	GV16, LR3, CV4, ST36	TH
Park (2003) [65]	SD	Male	6-OHDA	MA	GB34, LR3, LI4, LI11	Rotational test, TH, TrkB
Liang (2002) [66]	Wistar	Female	MFB transection	EA	GV14, GV21	TH, BDNF
Lin (2000) [67]	SD	Male, Female	6-OHDA	EA	LR3, SP6, ST36, GB34	DA, HVA, DOPAC
He (1998) [68]	SD	Male, Female	6-OHDA	EA	GV20, GV14	DA, NA, 5HT
Zhu (1996) [69]	C57BL/6	Male	MPTP	MA	GV20	DA, DOPAC

*Abbreviations*: *BDNF* Brain-derived neurotrophic factor, *BV* Bee-venom acupuncture, *CAT* Catalase, Caspase-3: caspase protein, *COX-2* Cyclooxygenase-2, *DA* Dopamine, *DAT* Dopamine active transporter, *DOPAC* Dihydroxyphenyl acetic acid, *EA* Electro-acupuncture, *Fn* Ferritin, *GABA* gamma-aminobutyric acid, *GDNF* Glial cell-derived neurotrophic factor, *GFAP* Glial fibrillary acidic protein, *GSH* Glutathione, *GSHpx* Glutathione peroxidase, *HSP70* 70 kilo Dalton heat shock proteins, *HVA* Homovanillic acid, *IL-1β* Interleukin-1 beta, *IL-6* Interleukin-6, *iNOS* Inducible nitric oxide synthase, *Lamp 1* Lysosomal-associated membrane protein 1, *MA* Manual acupuncture, *MAC-1* Macrophage-1 antigen, *MDA* Malondialdehyde, *NO* Nitric oxide, *nNos* Neuronal nitric oxide synthase, *MFB* Medial forebrain bundle, *MPTP* 1-methyl-4-phenyl-1,2,3,6-tetrahydropyridine, *p-p38 MARK* Phospho-p38 MAPK, *PCNA* Proliferating cell nuclear antigen, *SD* Sprague–Dawley, *SOD* Superoxide dismutase, *TH* Tyrosine hydroxylase, *TNF-α* Tumor necrosis factor alpha, *TrkB* Tropomyosin receptor kinase B, *UBE1* Ubiquitin-like Modifier Activating Enzyme 1, *UCH-L1* Ubiquitin C-terminal hydrolase, *5HIAA* 5-Hydroxyindoleacetic acid, *5HT* 5-hydroxytryptamine, *6-OHDA* 6-hydroxydopamine

### Animals of PD models

**Fig. 2:**
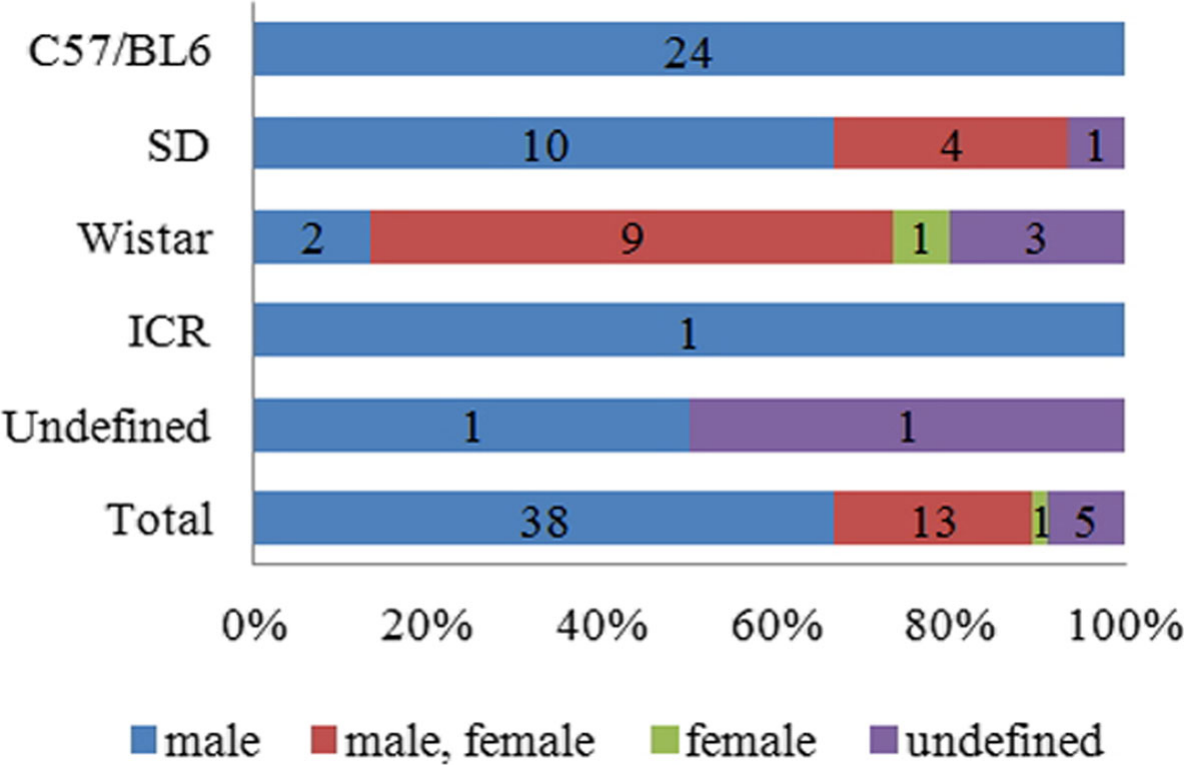
Sex differences according to the types of animal used as PD model

The animals of PD models included mice (C57/BL6 and ICR) and rats (Sprague–Dawley, and Wistar) (Fig. 2). The most frequently used animal PD model was C57/BL6, which was used in 24 articles, followed by SD and Wistar, each of which were used in 15 articles, and ICR and undefined animals, which were used in one article each. All of the studies using C57/BL6 animals used only males. Of the studies using SD animals, ten used males only, four used a male/female mix, and one used animals with undefined sex. Of the studies using Wistar animals, nine used a male/female mix, two used males only, three used animals with undefined sex, and one study used females only.

### Methods used to induce PD

**Fig. 3:**
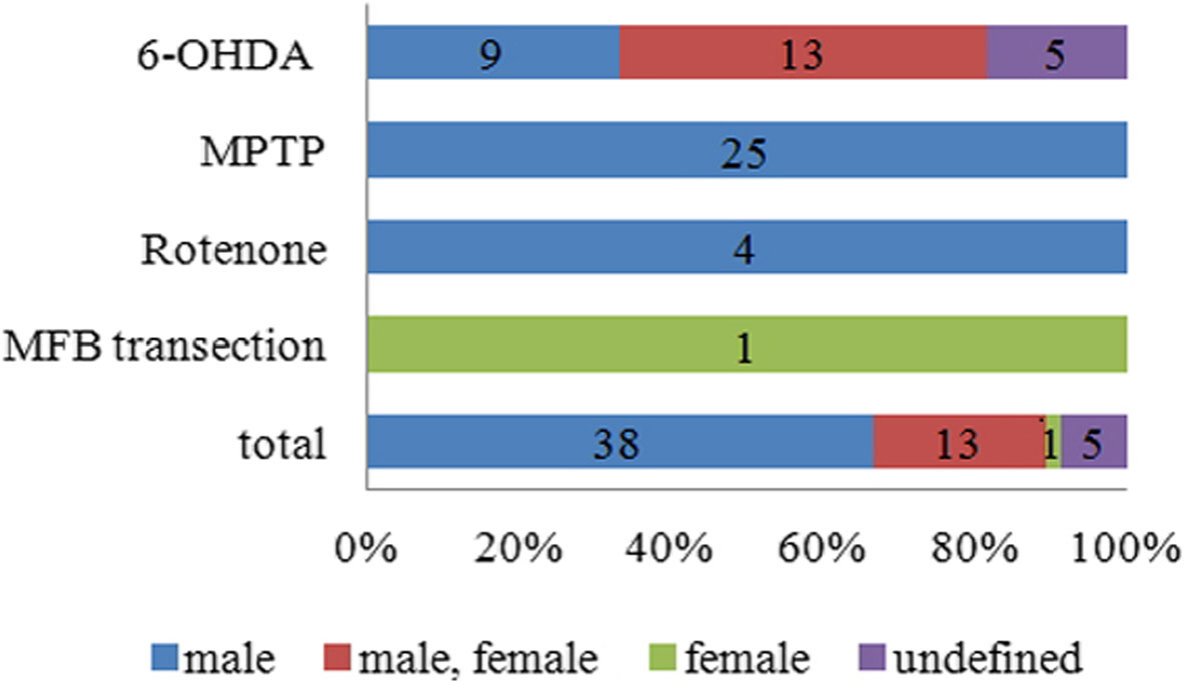
Sex differences according to the method used to induce PD

The drugs 6-hydroxydopamine (6-OHDA), 1-methyl-4-phenyl-1,2,3,6-tetrahydropyridine (MPTP), and rotenone, as well as medial forebrain bundle (MFB) transection, were used to induce PD in the animal models (Fig. 3). 6-OHDA was used in 47 % (27) of the studies, MPTP was used in 44 % (25) of the studies, and rotenone was used in 7 % (4) of the studies. MFB transection was used in 2 % (1) of the studies. Of the studies using 6-OHDA, 13 used a male/female mix, nine used only males, and five used animals with undefined sex. All of the studies using MPTP or Rotenone used only male animals. The study using MFB transection used only females. Therefore, three out of the four PD induction models studied were only used in animals of a single sex. Only the results of 6-OHDA induced animal PD models could potentially be compared between the sexes.

### Types of acupuncture

**Fig. 4:**
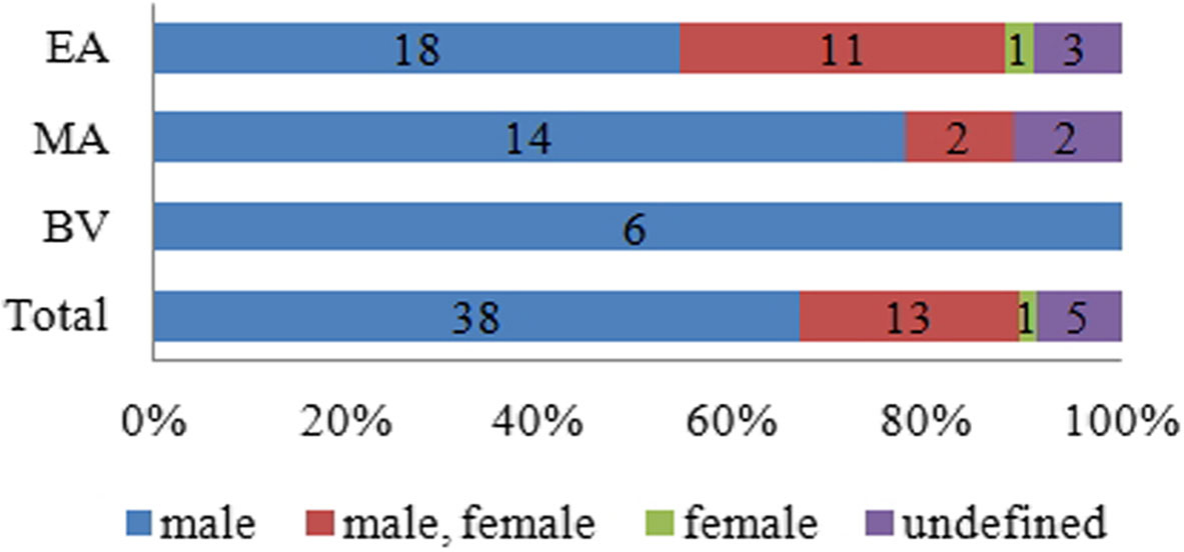
Sex differences according to the type of acupuncture performed

Electro-acupuncture (EA) was used in 54 % (38) of the studies, manual acupuncture (MA) was used in 30 % (18) of the studies, and bee-venom (BV) acupuncture was used in 11 % (6) of the studies. Of the studies using EA, 18 used only males, 11 used a male/female mix, three used animals of undefined sex, and one used only females. Of the studies using MA, 14 used only males, two used a male/female mix, and two used animals with undefined sex. All of the studies using BV acupuncture used only males (Fig. 4).

### Acupuncture points

Regardless of the type of acupuncture, the acupuncture points used consisted mainly of LR3, GB34, GV20, GV16, and ST36 (Additional file 1). LR3 was used in 35 % (20) of the studies, and GV34 and GV20 were each used in 26 % (16) of the studies. Of the studies using LR3, 14 used only males, three used a male/female mix, and three used animals with undefined sex. Of the studies using GB34, 14 used only males, and two used a male/female mix. Of the studies using GV20, eight used only males, seven used a male/female mix, and one used animals with undefined sex. Of the studies using GV16, seven used only males, three used a male/female mix, and three used animals with undefined sex. Of the studies using ST36, four used only males, two used animals with undefined sex, and one used a male/female mix.

### Behavioral test

Behavioral analyses were carried out using the rotational behavior test, the pole-climbing test, the swimming test, and locomotor counts (Additional file 2). The rotational behavior test was used in 56 % (10) of the studies, the pole-climbing test was used in 22 % (6) of the studies, and the swimming test, and locomotor counts were each used in 6 % (1) of the studies. The rotational behavior test was mainly used in conjunction with 6-OHDA (8 studies), the pole-climbing test was used in conjunction with MPTP (6 studies), and the swimming test and locomotor counts were each used in conjunction with MPTP (Additional file 3). The rotational behavior test was used in five studies with only males, four studies with a male/female mix, and one study with animals with undefined sex. The studies using the pole-climbing test, the swimming test, and locomotor counts were each conducted with males only. Of all studies including behavioral analyses, 72 % (13) of the studies used only male animals, 22 % (4) used a male/female mix, and 6 % (1) used animals with undefined sex. In these studies, PD was induced using MPTP in 53 % (9) of the studies and 6-OHDA in 47 % (8).

### Evaluation of treatment effectiveness

The effectiveness of the treatment on PD was evaluated by levels of tyrosine hydroxylase (TH), dopamine (DA), dihydroxyphenyl acetic acid (DOPAC), homovanillic acid (HVA), superoxide dismutase (SOD), glutathione (GSH), and brain-derived neurotrophic factor (BDNF) (Additional file 4). TH was the most frequently used method to determine the effectiveness of the treatment on PD (56 % [32] of the studies). Of the studies using TH, 26 used only males, two used a male/female mix, three used animals with undefined sex, and one used only females. Of the studies using DA, ten used only males, five used a male/female mix, and two used animals with undefined sex. Of the studies using DOPAC, seven used only males, and two used a male/female mix. Of the studies using HVA and GSH, respectively, four of each used only males, and two of each used a male/female mix. Of the studies using SOD, four used only males, and two used a male/female mix. Of the studies using BDNF, four used only males, one used a male/female mix, and one used only females.

## Discussion

We analyzed sex differences among previous studies that used animal PD models of acupuncture treatment. A total of 810 potentially relevant articles were identified, 57 of which met our inclusion criteria. C57/BL6 mice were the most frequently used (42 %) animal PD models. Most of the studies evaluating the effectiveness of acupuncture treatment for PD were performed using only male animals (67 %); only one study (2 %) was performed using female animals.

Many studies have inadvertently excluded females from animal studies of acupuncture treatment for PD. Kang et al. suggested that acupuncture could be used as a neuroprotective intervention for inhibiting microglial activation and inflammatory events in the MPTP-induced male PD model [52]. Yu et al. showed that acupuncture treatment displays antioxidative and/or neuroprotective properties in the 6-OHDA lesioned male rat PD models [3]. Although a few studies were performed using a male/female mix, they could not combine and compare the results from male versus female animals. Only one report used female animals, in which was a study in which different frequencies of chronic EA stimulation were tested in a partially-lesioned female rat model of PD induced by transection of the MFB. This study suggested that long-term high frequency EA is effective in halting the degeneration of dopaminergic neurons in the substantia nigra (SN). Because the studies of male PD models generated using MFB transection are nonexistent, we could not compare the sex differences in this model. Taken together, there is currently insufficient evidence from past studies to determine whether there are sex differences in the effectiveness of acupuncture for animal PD models. In the future, studies should be performed using a male/female mix to minimize performance bias, and ideally should include a comparison of the sex differences.

Animal studies have often focused primarily on males. For the most part, examination of the differences between males and females has been disregarded in biomedical research, leaving gaps in our knowledge [42]. Recently, new drugs have been developed without considering the physiological characteristics of females or sex differences. Women have therefore been frequently exposed to dangerous side effects because the experimental studies and clinical trials had mainly used male subjects [70]. The lack of female participation in drug-development studies affects males as well as females; when side effects not seen in males during the drug safety checks appear in females, the approval of the drugs is delayed, and male patients waiting for the drugs consequently suffer. The NIH requires applicants to report their cell and animal inclusion plans as part of the preclinical experimental design. Despite this NIH policy, numerous scientific publications continue to neglect sex-based considerations and analyses in preclinical and clinical research. A stronger commitment to reporting sex-specific results will strengthen the evidence base [13]. Fortunately, sex differences are increasingly recognized as factors that influence the incidence and disease manifestations of all diseases, including neurodegenerative disorders.

Some gender differences have been documented for PD [3, 4]. Paven et al. suggested gender differences in the epidemiology, clinical features, treatment outcomes (medical and surgical/deep brain stimulation), and social impact among all available PD studies [4]. Wooten et al. performed a meta-analysis of the differences in the incidence of PD between men and women [3]. Smith et al. summarized evidence that estrogen and selective estrogen receptor modulators are neuroprotective in PD, and reviewed sex differences in basal ganglia function and dopaminergic pathways [71, 72]. Consistent with these past studies, if acupuncture research involved both males and females, additional studies of acupuncture for PD would provide a more robust conclusion about sex differences in this treatment.

### Review limitations and future areas of research

A number of gaps in the reviewed literature were identified in relation to study quality and findings. Study quality could be improved by using female animal models because they reflect the physiological characteristics of both males and females to fully evaluate the effectiveness and safety of the treatment for each sex, which is largely missing in the literature so far.

## Conclusions

The results of our review suggest that acupuncture is an effective treatment for animal PD models, but there is insufficient evidence to determine whether sex differences exist in response to this treatment. Future studies should examine the effects of acupuncture in animal PD models of both sexes, to reflect the physiological characteristics of females as well as males, and to fully evaluate the effect and safety of this treatment.

## Additional files

Additional file 1: Sex differences according to the acupuncture points used. (TIF 781 kb)
Additional file 2: Sex differences according to behavioral tests used. (TIF 776 kb)
Additional file 3: Behavioral tests performed categorized by the method used to induce PD. (TIF 638 kb)
Additional file 4: Sex differences according to the method of evaluation of treatment effectiveness. (TIF 842 kb)

## Abbreviations

BDNF: Brain-derived neurotrophic factor
BV: Bee-venom acupuncture
DA: Dopamine
DOPAC: Dihydroxyphenyl acetic acid
EA: Electro-acupuncture
GSH: Glutathione
HVA: Homovanillic acid
MA: Manual acupuncture
MFB: Medial forebrain bundle
MPTP: 1-methyl-4-phenyl-1,2,3,6-tetrahydropyridine
SD: Sprague-Dawley
SOD: Superoxide dismutase
TH: Tyrosine hydroxylase
6-OHDA: 6-hydroxydopamine
