# Supplementary figures and images for: Sex differences in acupuncture effectiveness in animal models of Parkinson's disease: a systematic review
Source: BMC Complement Altern Med. 2016 Nov 3;16:430. doi: 10.1186/s12906-016-1405-5 (PMC5094083; doi:10.1186/s12906-016-1405-5)

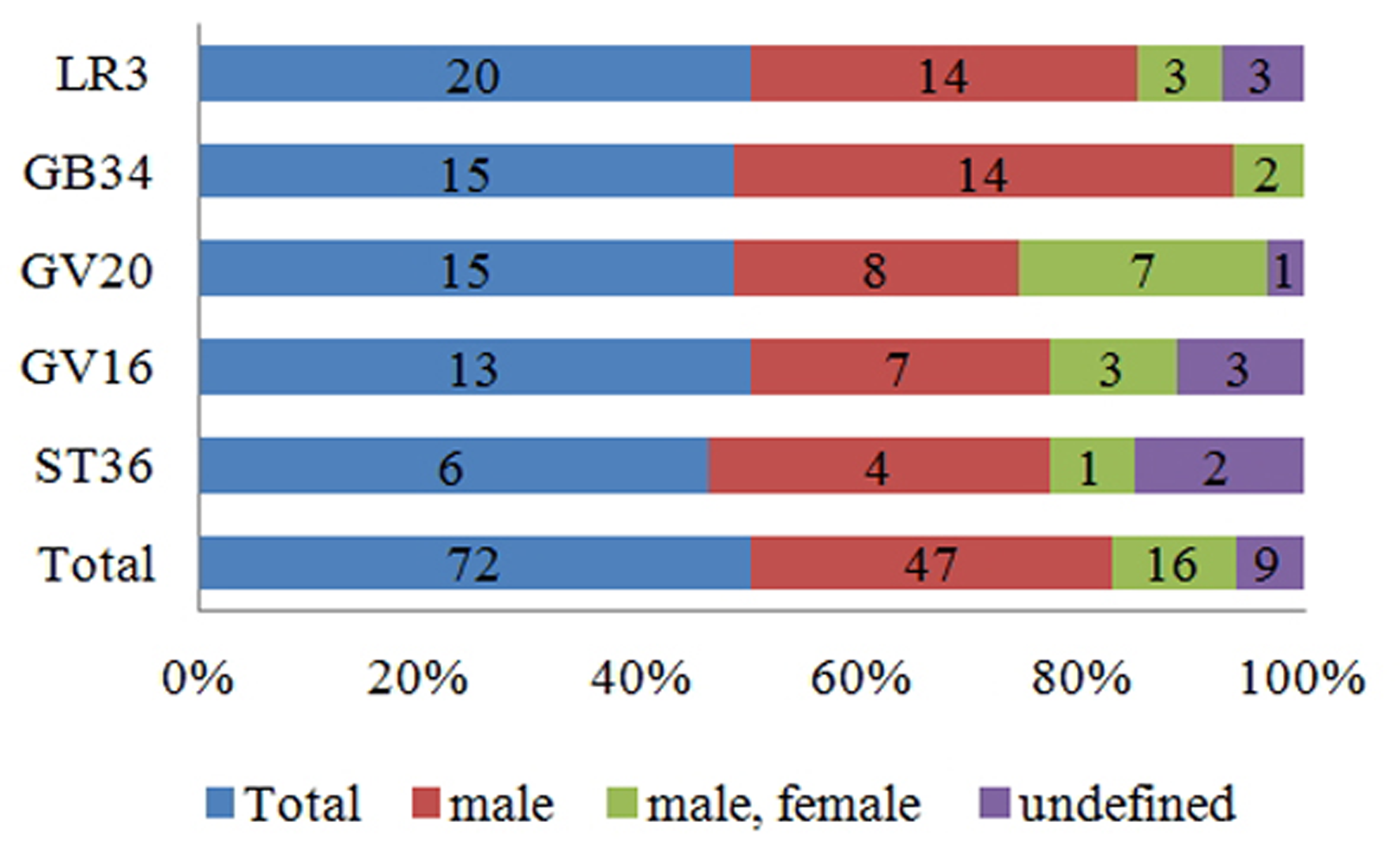

Supplement: Additional file 1: — Sex differences according to the acupuncture points used. (TIF 781 kb) [file 12906_2016_1405_MOESM1_ESM.tif]

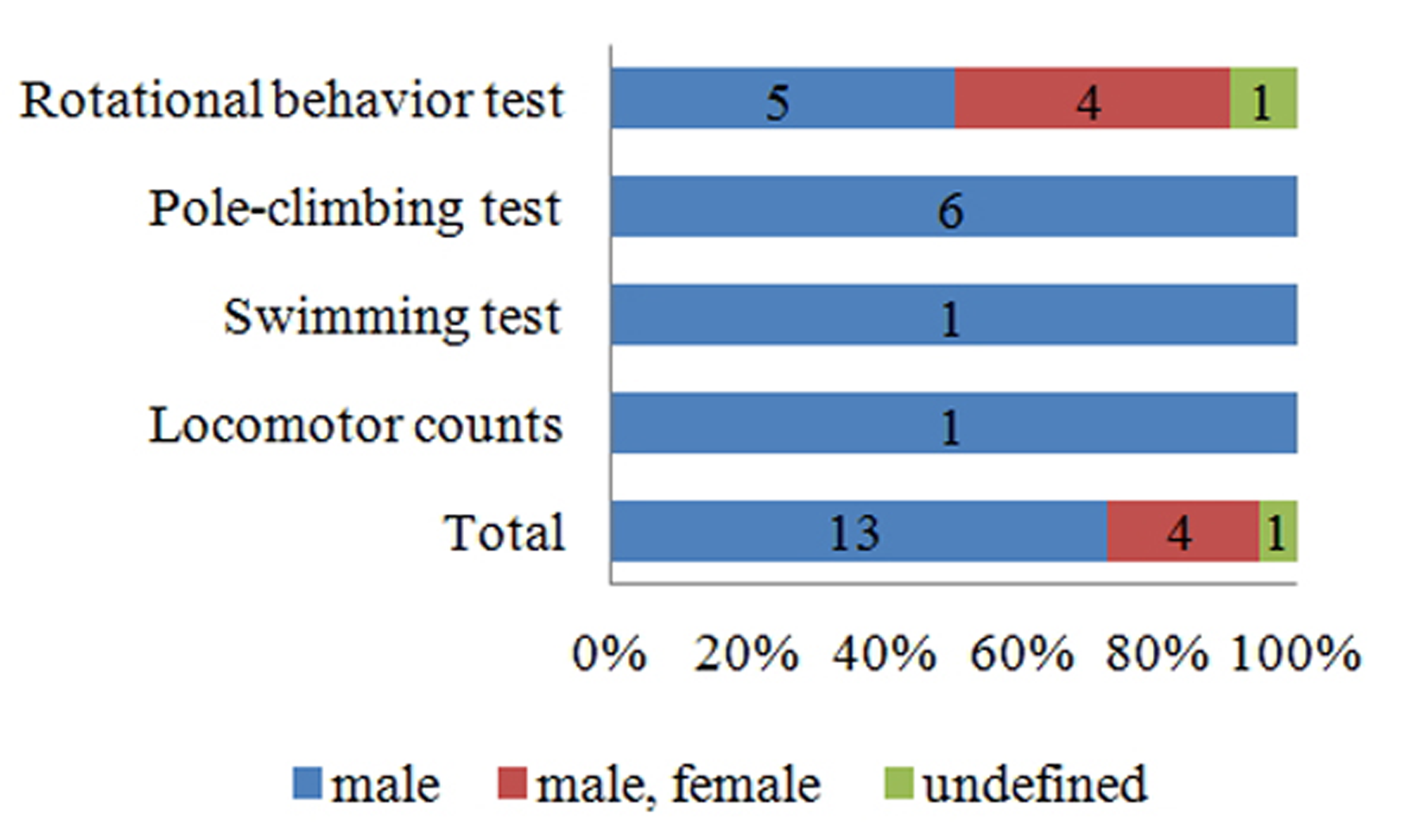

Supplement: Additional file 2: — Sex differences according to behavioral tests used. (TIF 776 kb) [file 12906_2016_1405_MOESM2_ESM.tif]

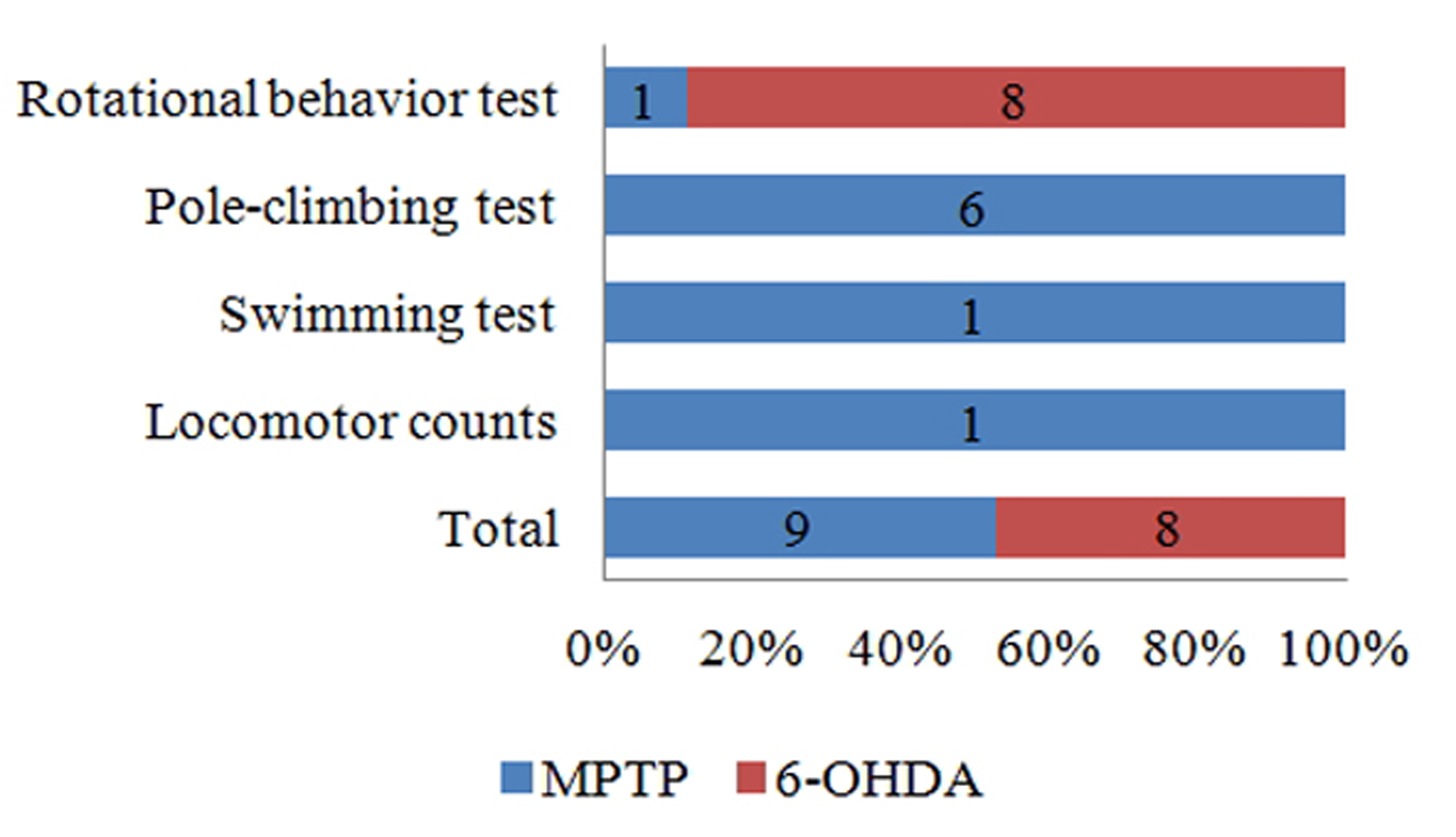

Supplement: Additional file 3: — Behavioral tests performed categorized by the method used to induce PD. (TIF 638 kb) [file 12906_2016_1405_MOESM3_ESM.tif]

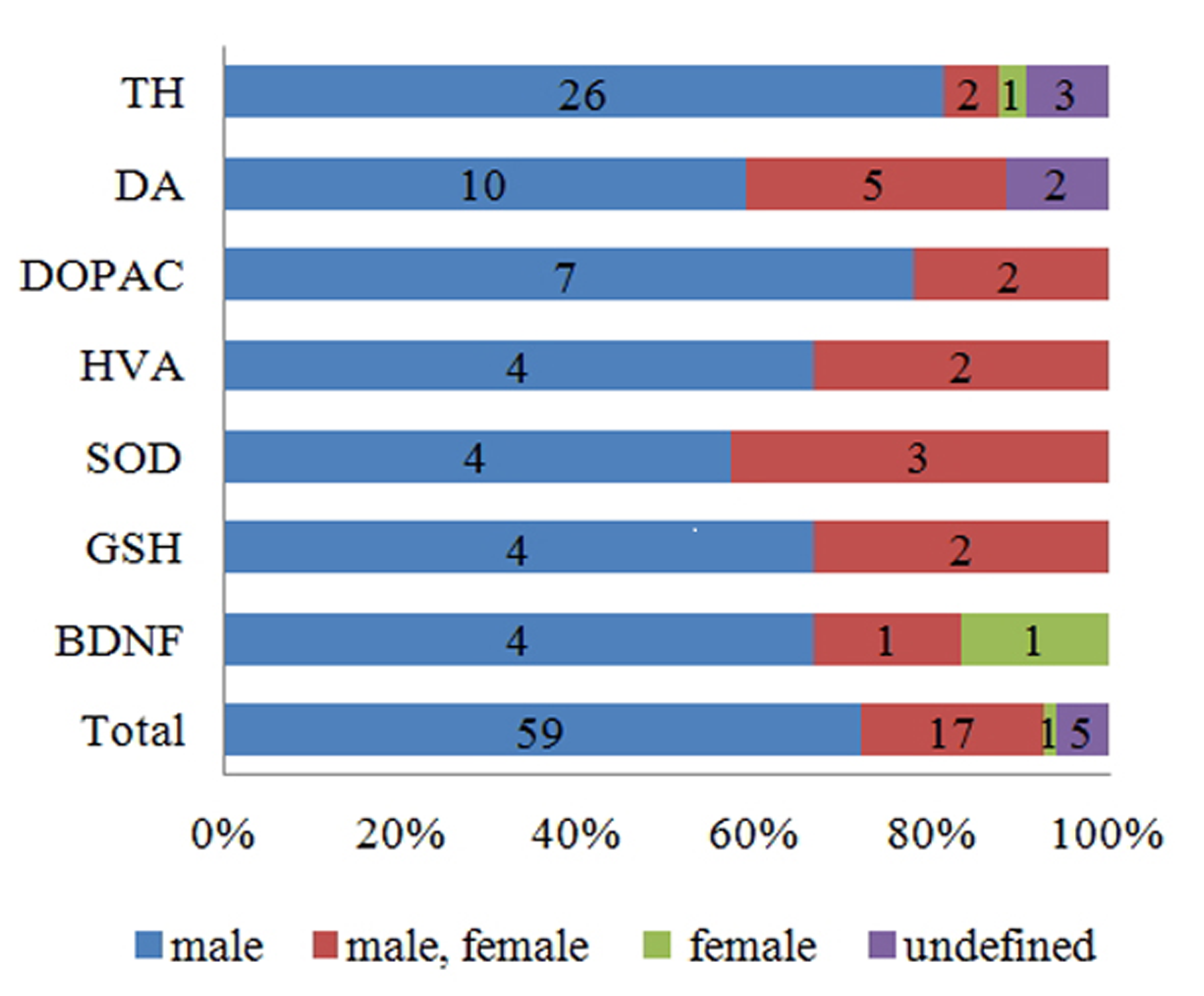

Supplement: Additional file 4: — Sex differences according to the method of evaluation of treatment effectiveness. (TIF 842 kb) [file 12906_2016_1405_MOESM4_ESM.tif]
